# Supplementary material for: Selective inhibition of the amyloid matrix of Escherichia coli biofilms by a bifunctional microbial metabolite
Source: NPJ Biofilms Microbiomes. 2023 Oct 19;9:81. doi: 10.1038/s41522-023-00449-6 (PMC10587114; doi:10.1038/s41522-023-00449-6)
Supplement: Supplementary file 1 — Supplementary Information [file 41522_2023_449_MOESM1_ESM.pdf]

Supplementary Information
for

Selective inhibition of the amyloid matrix of *Escherichia coli* biofilms by a bifunctional microbial metabolite

Estefanía Cordisco[1], María Inés Zanor[2], Diego Martín Moreno[3] and Diego Omar Serra[1*]

[1] Laboratorio de Estructura y Fisiología de Biofilms Microbianos, Instituto de Biología Molecular y Celular de Rosario (IBR, CONICET-UNR), Predio CONICET Rosario, Ocampo y Esmeralda, (2000) Rosario, Argentina.

[2] Laboratorio de Metabolismo y Señalización en Plantas, Instituto de Biología Molecular y Celular de Rosario (IBR, CONICET-UNR), Predio CONICET Rosario, Ocampo y Esmeralda, (2000) Rosario, Argentina.

[3] Instituto de Química Rosario (IQUIR, CONICET-UNR), Predio CONICET Rosario, Ocampo y Esmeralda, (2000) Rosario, Argentina. Facultad de Ciencias Bioquímicas y Farmacéuticas, Suipacha 531, (2000) Rosario, Argentina.

* Address correspondence to Diego O. Serra: dserra@ibr-conicet.gov.ar

Content

- Supplementary Figures 1-4
- Supplementary Methods
- Supplementary References
- Source Data (uncropped scans of western blots and gels)

Supplementary Figure 1

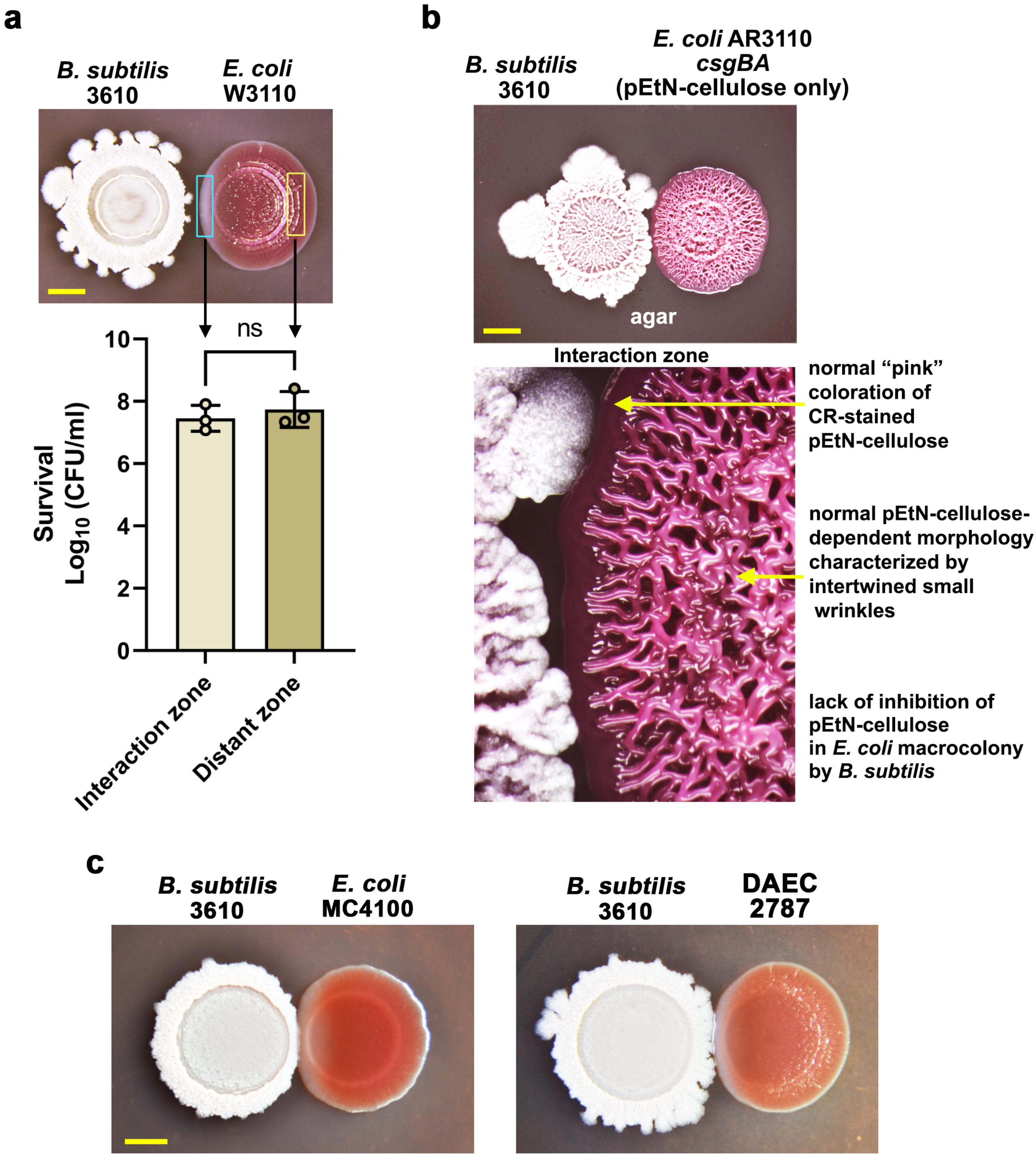

Supplementary Figure 1. Effects of *B. subtilis* on *E. coli* viability, on pEtN-cellulose production and on amyloid curli production in macrocolonies of other *E. coli* strains. (a) Graph showing the number of *E. coli* viable cells at the zone of interaction or at a zone distant from *B. subtilis*. Cell biomass from each zone -as boxed in the image presented above the graph- was collected, resuspended and adjusted to the same OD_{578} and subjected to viable cell counting. The data are means $\pm$ standard deviations of colony forming unit (CFU) counts derived from the analysis of three independent interactions. ns, no significant difference (two-tailed unpaired t-tests). **(b)** Macrocolony biofilms of *E. coli* AR3110 Δ*csgBA* grown on CR-containing salt-free LB agar in close interaction with a macrocolony of *B. subtilis* 3610. The Δ*csgBA* AR3110 macrocolony exhibits small intertwined wrinkles and a CR-based pink coloration characteristic of pEtN-cellulose. The images reveal that *B. subtilis* 3610 does not affect the morphological and CR-staining patterns of the Δ*csgBA* AR3110 macrocolony. **(c)** Macrocolony biofilms of *E. coli* MC4100 and Diffusely Adhering *E. coli* (DAEC) 2787 grown on CR-containing salt-free LB agar in close interaction with a macrocolony of *B. subtilis* 3610. Images show that the inhibitory effect of *B. subtilis* on curli is reproduced in macrocolonies of other *E. coli* strains. Scale bars in (a, b, and c) represent 5 mm.

Supplementary Figure 2

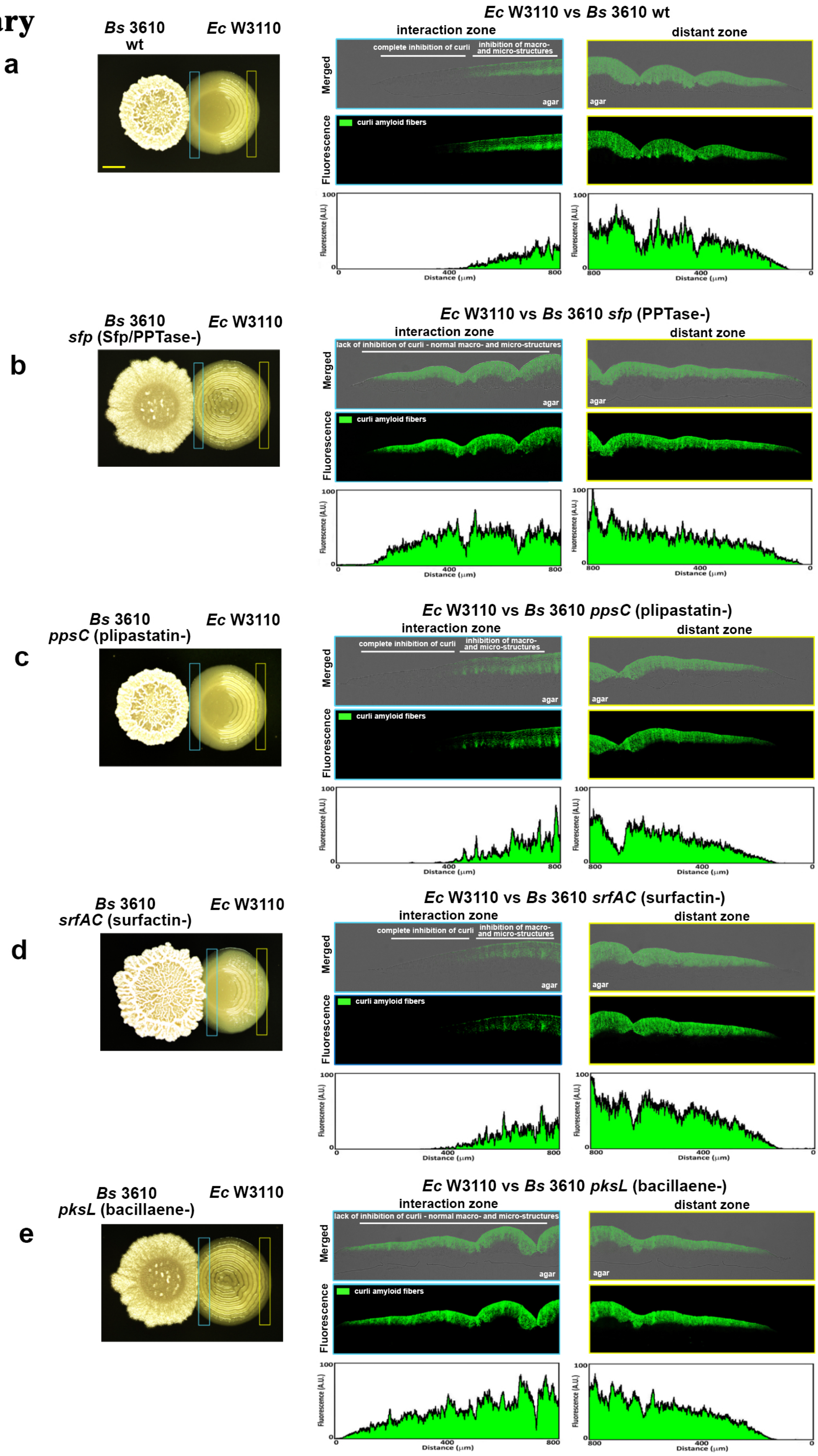

Supplementary Figure 2. Effect of Sfp-dependent secondary metabolites of *B. subtilis* 3610 on curli production and colony morphology examined at the microscale. (a-e) Macrocolony biofilms of *E. coli* W3110 grown on TS-containing salt-free LB agar in interaction with *B. subtilis* 3610 wt or derivative strains deficient in the phosphopantetheinyl transferase (*sfp*), surfactin (*srfAC*), plipastatin (*ppsC*) or bacillaene (*pksL*) (Left-hand side images). Fluorescence and merged fluorescence/phase contrast images of representative cross-sections through the W3110 macrocolony at zones of interaction or distant from respective *B. subtilis* strains (Right-hand side images). The spectral plots depict quantified patterns of TS fluorescence in respective cross-sections. Data provides further visual details demonstrating that in the absence of bacillaene only, curli production and hence the curli-dependent colony morphology occur normally. Scale bar in (a) represents 5 mm and is applicable to all macrocolony images presented in (b, c, d, and e).

Supplementary Figure 3

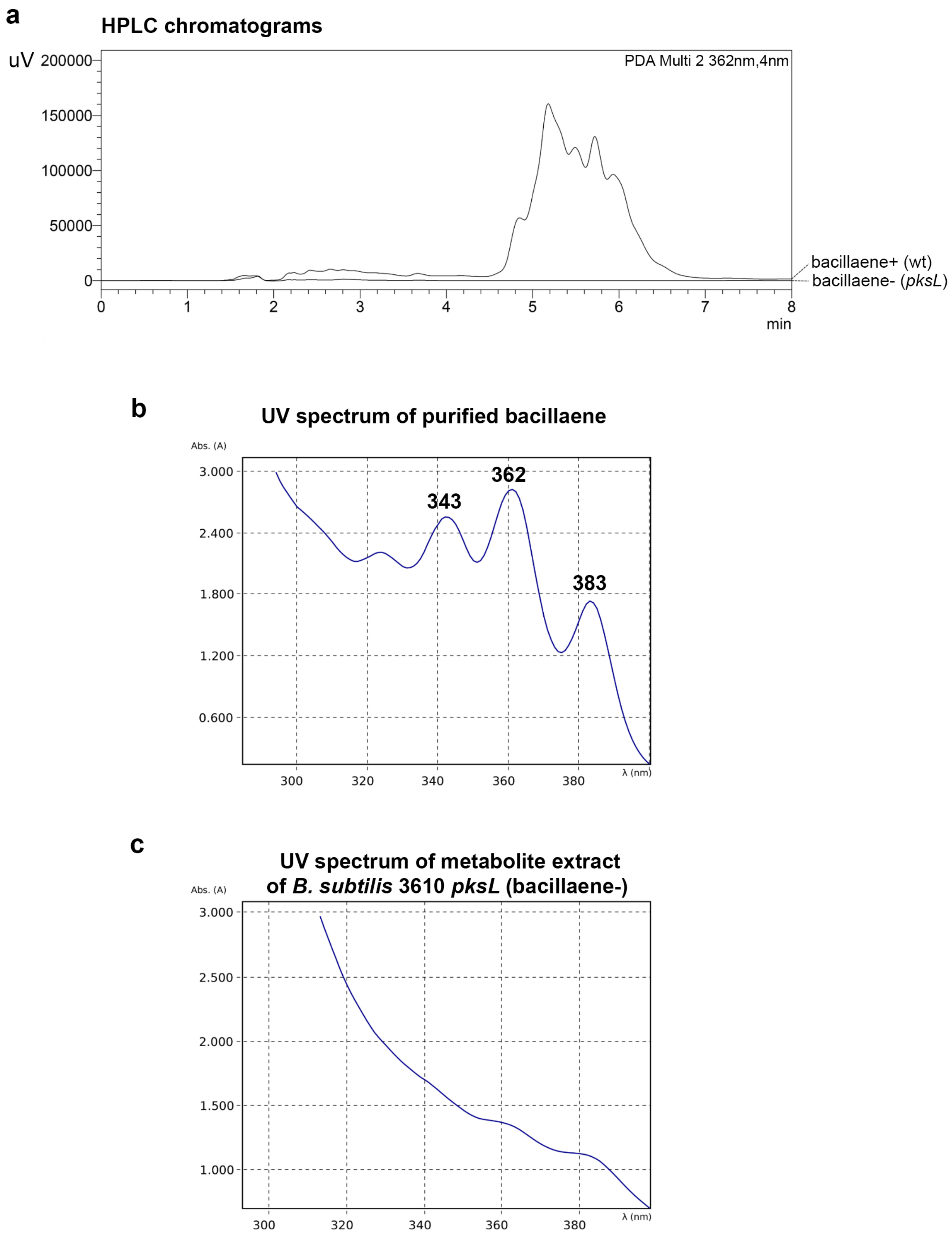

Supplementary Figure 3. **Bacillaene purification. (a)** HPLC chromatograms at 362 nm showing bacillaene peaks in the processed *B. subtilis* wt extract, absent in the metabolite extract from the *pksL* mutant. **(b-c)** Absorption UV spectra of purified bacillaene fraction (b) and of metabolite extract from the *pksL* mutant (c). The UV spectrum in (b) shows the characteristic band pattern of bacillaene, with absorption peaks appearing at 343, 362 and 383 nm, as previously reported (reference 21 in article). Consistent with this, bacillaene absorption bands are absent in spectrum shown in (c).

Supplementary Figure 4

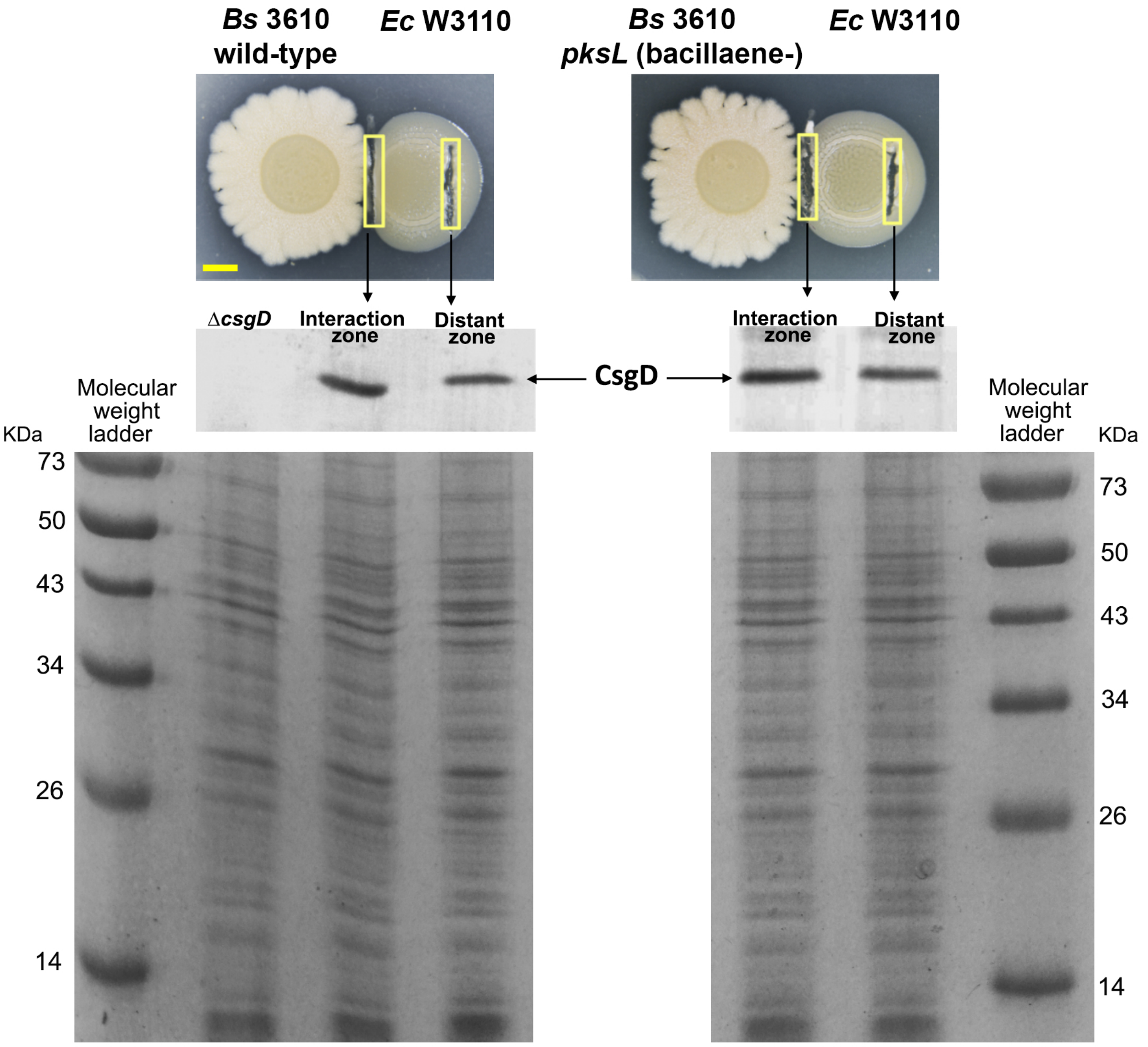

Supplementary Figure 4. Sample loading control for western blot analysis of the cellular level of CsgD in W3110 macrocolonies interacting with *B. subtilis* 3610 wild type or *pksL* mutant, as depicted in Figure 4. *E. coli* biomass from both the interaction and distant zones - as shown in the images - was collected and subjected to western blot analysis using specific antibodies targeting CsgD. The bottom images display the respective samples loaded onto the SDS-PAGE gels. Scale bar represents 5 mm.

Supplementary Methods

Time-lapse imaging of interacting macrocolony biofilms

Interacting *E. coli-B. subtilis* macrocolonies were set by inoculating 5 µl of overnight cultures of each strain onto agar with a separation distance of 1.5 cm. Macrocolonies were allowed to grow in an incubation chamber conditioned to maintain constant temperature (28°C) and constant humidity to avoid agar dehydration. The chamber was additionally equipped with LED illumination, a stage with black background to place the agar plate and mechanical arms to hold and adjust the position of a D5300 reflex camera (Nikon) used for imaging. D5300 camera was equipped with a Dx-Vr AF-P Nikkor18-55 mm objective (Nikon) and a 12-mm Macro extension tube for macro photography. Images of the developing colonies were taken every 10 min over a 4-day period using the “Internal timer shooting” function of the camera. Images were then processed and adjusted in batch mode using Photoshop CC 2020 (Adobe) and assembled to create time-lapse movies using Windows movie maker (Microsoft). Timer and scale were inserted into the movies with Fiji (ImageJ) software.

Bacillaene extraction and purification

The extraction and purification of bacillaene was performed as previously described by [1] with modifications. All procedures were carried out minimizing light exposition of the samples. One-liter cultures of *B. subtilis* 3610 wt (or *pksL* mutant, included as control) in salt-free LB broth were set up at an initial OD_{578} of 0.05 and incubated for 20 h at 28°C under shaking conditions. Cells were removed by centrifugation (8,000 rpm, 20 min, 25°C) and subsequent filtration (bottle top vacuum filter, 0.22 µm, Millipore). Each cell-free supernatant was then freeze-dried; the residue resuspended in 50 ml water and supplemented with one volume of ethyl acetate for bacillaene extraction. The mixture was incubated under agitation for 30 min at 28°C, followed by a 15-min-incubation at rest to allow the phases to equilibrate. The bacillaene-containing organic phase was recovered and subjected to evaporation under reduced pressure. The residue was the dissolved in methanol and filtered through 0.22 µm filters (Whatman, GE Healthcare). The sample was then chromatographed on a Shimadzu LC-2050C 3D high-pressure liquid chromatograph (HPLC) as follows. The filtered sample was injected (sequential 10 µl injections) onto a Hypersil ODS C18 column (150 x 4.6 mm i.d., 3 µm particle size, ThermoFisher) using 20 mM NaPi and acetonitrile as mobile phases A and B, respectively. The elution profile was 0-2 min 35% B, 2-8 min 35-40% B, 8-10 min 40% B, 10-12 40-35% B, 12-15 min 35% B. The mobile phase flow rate was 1 mL/min. Absorbance was monitored at 362 nm using a photo diode (PDA) array. Correspondence of peaks to bacillaene was corroborated

by comparatively analyzing the extract from the *pksL* mutant devoid of bacillaene. The fraction containing exclusively bacillaene peaks was evaporated under reduced pressure. The residue was weighed and dissolved in methanol to achieve a bacillaene concentration of 700 μg/ml. Photostability of bacillaene was evaluated by UV spectrophotometry.

Purification of soluble CsgA protein

Overexpression and non-denaturing purification of C-terminal His6-tagged CsgA from bacterial supernatant was performed as previously described with some modifications [2]. Briefly, *E. coli* LSR12 (Δ*csgDEFG/csgBAC*) freshly co-transformed with pMC3/pMC1 was grown with shaking at 37°C to an OD_{578} of 1, IPTG (0.25 mM final concentration) was added to induce CsgA production, and the cultures were incubated for additional 2 h at 37°C. Cells were pelleted by centrifugation and the filtered supernatant was stored overnight at 4°C under shaking. The purification steps were performed at 4°C and the elutes and samples were stored on ice. The filtered supernatant was flowed over a 2.5 × 10 cm column packed with 4 ml of Ni-NTA and the column was washed with KPi buffer (50 mM potassium phosphate buffer, pH 7.2). CsgA was eluted from the column using 0.1 M imidazole in KPi buffer and 0.5 M imidazole in KPi buffer in the final elution step. Fractions were collected and analyzed for the presence of protein by UV_{280} using the BioSpectrometer (Eppendorf). The combined fractions were loaded onto PD10 desalting columns (GE Healthcare), eluted with 3.5 ml KPi buffer and filtered through 0.02 μm anotop syringe filters (Whatman, GE Healthcare). The final concentration was measured by the Sedmak and Grossberg method [3]. Samples were then immediately used for *in vitro* polymerization assays.

Scanning electron microscopy

Amyloid fibers formed by CsgA polymerization *in vitro* in KPi buffer were subjected to dehydration in a graded alcohol series (30, 50, 70, 90, and 100% ethanol) and dried in a EM CPD 300 critical point drier (Leica). The dried material was mounted on an aluminum stub using double-sided adhesive tape and coated with gold in a sputter coater [4]. The fibers were examined with a Quanta 200 FEG Scanning Electron Microscope (FEI) operating at an accelerating voltage of 5 kV under high vacuum mode.

SUPPLEMENTARY REFERENCES

1 Muller, S. *et al.* Bacillaene and sporulation protect *Bacillus subtilis* from predation by *Myxococcus xanthus*. *Appl Environ Microbiol* **80**, 5603-5610, doi:10.1128/AEM.01621-14 (2014).

2 Zhou, Y., Smith, D. R., Hufnagel, D. A. & Chapman, M. R. Experimental manipulation of the microbial functional amyloid called curli. *Methods Mol Biol* **966**, 53-75, doi:10.1007/978-1-62703-245-2_4 (2013).

3 Sedmak, J. J. & Grossberg, S. E. A rapid, sensitive, and versatile assay for protein using Coomassie brilliant blue G250. *Analytical biochemistry* **79**, 544-552, doi:10.1016/0003-2697(77)90428-6 (1977).

4 Serra, D. O., Richter, A. M., Klauck, G., Mika, F. & Hengge, R. Microanatomy at cellular resolution and spatial order of physiological differentiation in a bacterial biofilm. *mBio* **4**, e00103-00113, doi:DOI: 10.1128/mBio.00103-13 (2013).

Source Data
Uncropped and unprocessed scans of Western blots and gels

Scans of uncropped Western blots presented in Figure 4e

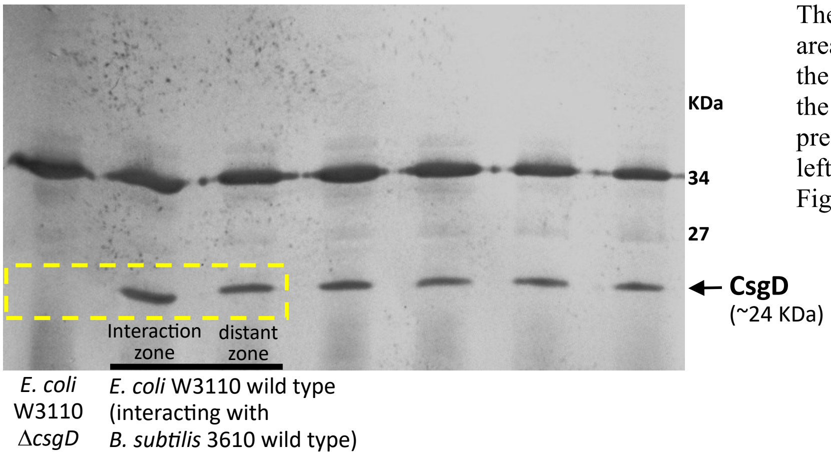

The yellow dashed area corresponds to the cropped part of the western blot presented on the left-hand side of Figure 4e.

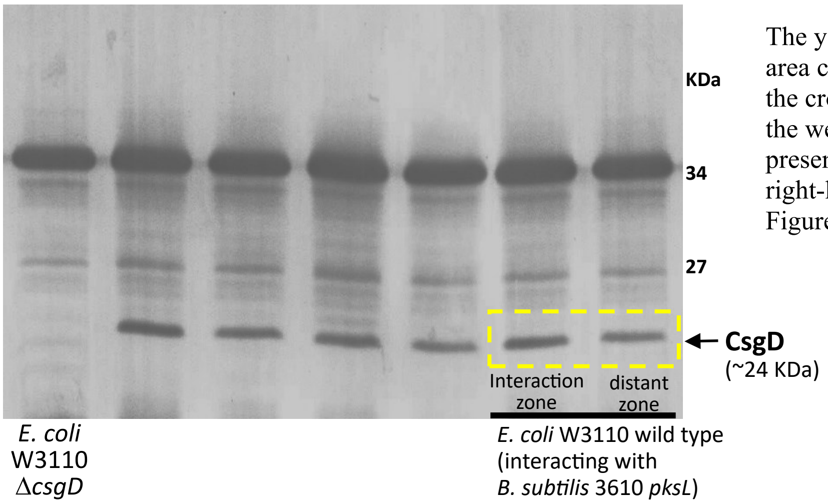

The yellow dashed area corresponds to the cropped part of the western blot presented on the right-hand side of Figure 4e.

Scan of uncropped Western blot presented in Figure 5

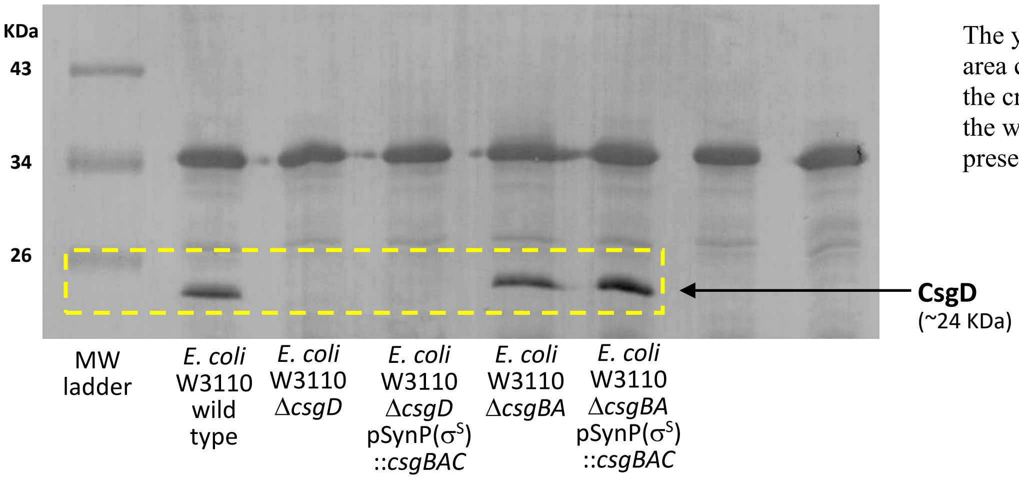

The yellow dashed area corresponds to the cropped part of the western blot presented in Figure 5.

Scan of uncropped Western blot presented in Figure 6a

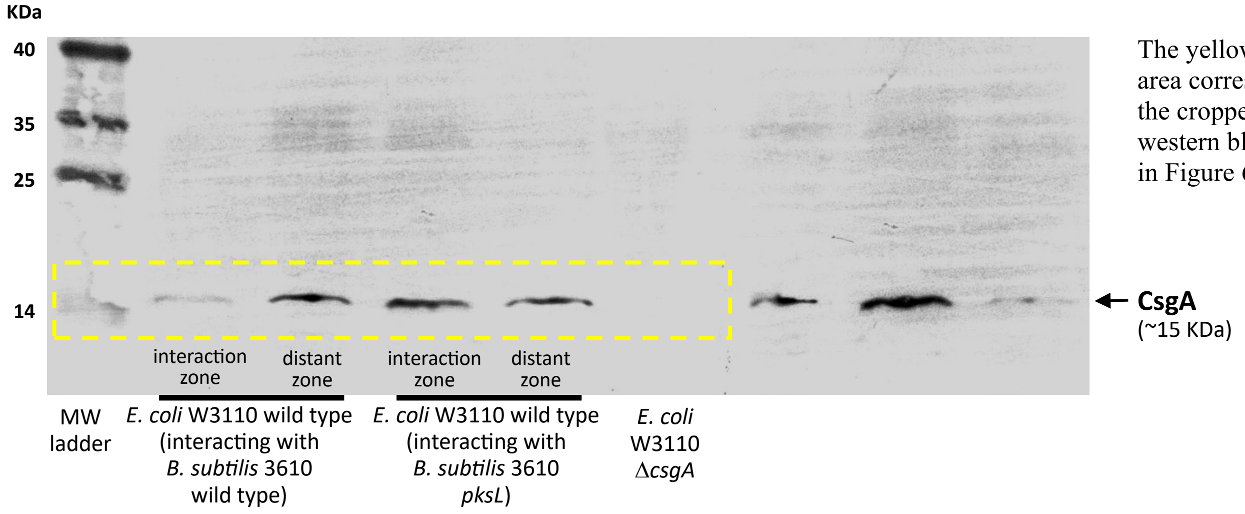

The yellow dashed area corresponds to the cropped part of the western blot presented in Figure 6a.

Scan of uncropped Gel presented in Figure 6a

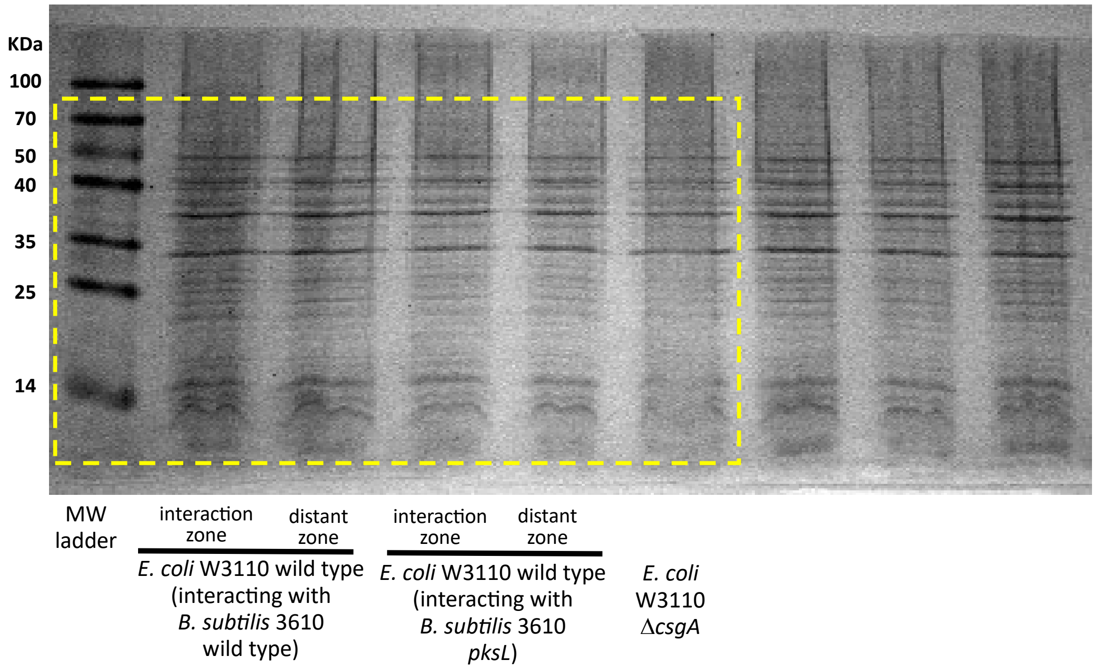

The yellow dashed area corresponds to the cropped part of the gel presented in Figure 6a.

Scan of uncropped Western blot presented in Figure 6b

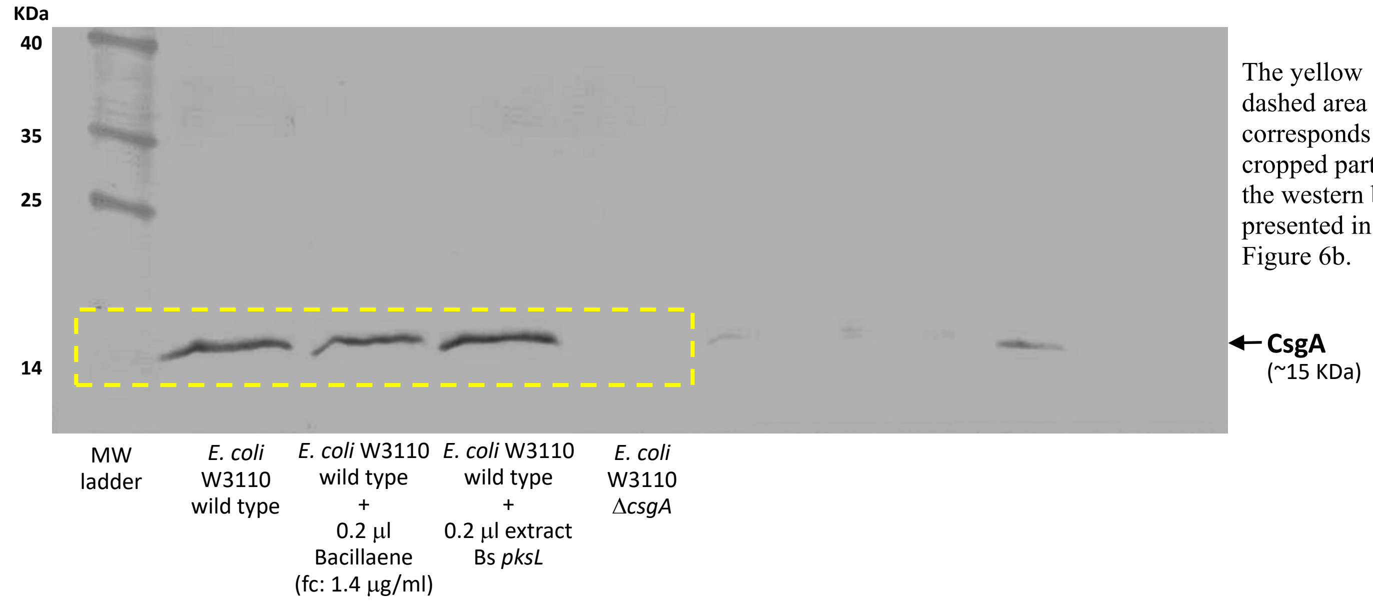

The yellow dashed area corresponds to the cropped part of the western blot presented in Figure 6b.

Scan of uncropped gel presented in Figure 6b

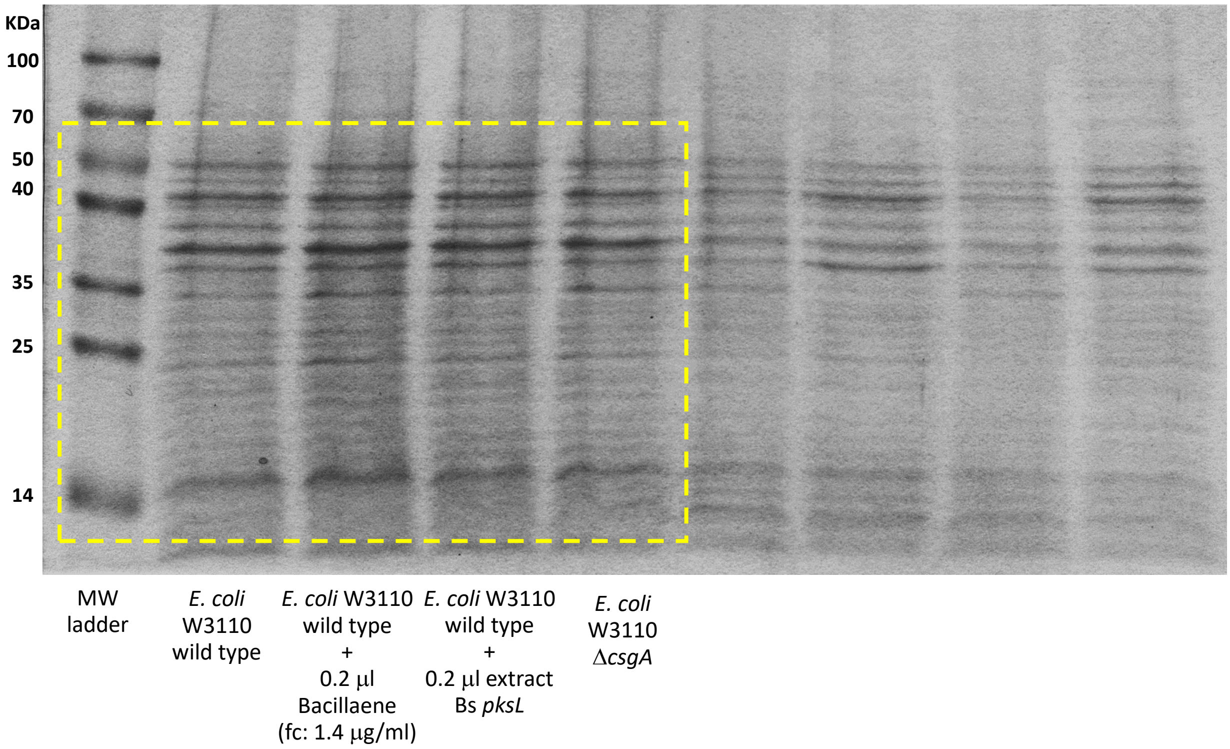

The yellow dashed area corresponds to the cropped part of the gel presented in Figure 6b.
