# Supplementary material for: Selective inhibition of the amyloid matrix of Escherichia coli biofilms by a bifunctional microbial metabolite
Source: NPJ Biofilms Microbiomes. 2023 Oct 19;9:81. doi: 10.1038/s41522-023-00449-6 (PMC10587114; doi:10.1038/s41522-023-00449-6)
Supplement: Supplementary file 2 — Reporting Summary [file 41522_2023_449_MOESM2_ESM.pdf]

nature portfolio

Corresponding author(s): Diego Omar Serra

Last updated by author(s): Sep 25, 2023

Reporting Summary

Nature Portfolio wishes to improve the reproducibility of the work that we publish. This form provides structure for consistency and transparency in reporting. For further information on Nature Portfolio policies, see our Editorial Policies and the Editorial Policy Checklist.

Statistics

For all statistical analyses, confirm that the following items are present in the figure legend, table legend, main text, or Methods section.

n/a	Confirmed	
☐	☒	The exact sample size (*n*) for each experimental group/condition, given as a discrete number and unit of measurement
☐	☒	A statement on whether measurements were taken from distinct samples or whether the same sample was measured repeatedly
☐	☒	The statistical test(s) used AND whether they are one- or two-sided<br>*Only common tests should be described solely by name; describe more complex techniques in the Methods section.*
☒	☐	A description of all covariates tested
☒	☐	A description of any assumptions or corrections, such as tests of normality and adjustment for multiple comparisons
☐	☒	A full description of the statistical parameters including central tendency (e.g. means) or other basic estimates (e.g. regression coefficient) AND variation (e.g. standard deviation) or associated estimates of uncertainty (e.g. confidence intervals)
☐	☒	For null hypothesis testing, the test statistic (e.g. *F*, *t*, *r*) with confidence intervals, effect sizes, degrees of freedom and *P* value noted<br>*Give P values as exact values whenever suitable.*
☒	☐	For Bayesian analysis, information on the choice of priors and Markov chain Monte Carlo settings
☒	☐	For hierarchical and complex designs, identification of the appropriate level for tests and full reporting of outcomes
☒	☐	Estimates of effect sizes (e.g. Cohen's *d*, Pearson's *r*), indicating how they were calculated

Our web collection on statistics for biologists contains articles on many of the points above.

Software and code

Policy information about availability of computer code

Data collection	Excel (Microsoft Office 2016, Microsoft) used to collect data from Synergy 2 microplate reader (BioTeK).
Data analysis	Fiji (open source image processing package based on ImageJ2) used for image analysis; Adobe Photoshop CC 2020 21.0.2 (Adobe) used for image analysis and figure creation; Windows movie maker (Microsoft) used for assembling images to create time-lapse movies; GraphPad Prism 9 (GraphPad Software) used for statistical analysis.

For manuscripts utilizing custom algorithms or software that are central to the research but not yet described in published literature, software must be made available to editors and reviewers. We strongly encourage code deposition in a community repository (e.g. GitHub). See the Nature Portfolio guidelines for submitting code & software for further information.

Data

Policy information about availability of data

All manuscripts must include a data availability statement. This statement should provide the following information, where applicable:

- Accession codes, unique identifiers, or web links for publicly available datasets
- A description of any restrictions on data availability
- For clinical datasets or third party data, please ensure that the statement adheres to our policy

The data supporting the findings of this study are available within the article and its Supplementary Information file, including uncropped and unprocessed scans of western blots and gels. Other unprocessed data supporting the findings of this study are available from the corresponding author upon reasonable request.

Research involving human participants, their data, or biological material

Policy information about studies with human participants or human data. See also policy information about sex, gender (identity/presentation), and sexual orientation and race, ethnicity and racism.

Reporting on sex and gender	Our study is situated within the field of microbiology. It does not involve human participants, human data, or any biological material from humans or animals.
Reporting on race, ethnicity, or other socially relevant groupings	Our study does not involve any race, ethnicity or other socially relevant grouping. Our study does not involve human participants, human data, or any biological material from humans or animals.
Population characteristics	Our study does not involve human participants, human data, or any biological material from humans or animals.
Recruitment	Our study does not involve human participants, human data, or any biological material from humans or animals.
Ethics oversight	Our study does not involve human participants, human data, or any biological material from humans or animals.

Note that full information on the approval of the study protocol must also be provided in the manuscript.

Field-specific reporting

Please select the one below that is the best fit for your research. If you are not sure, read the appropriate sections before making your selection.

☒ Life sciences ☐ Behavioural & social sciences ☐ Ecological, evolutionary & environmental sciences

For a reference copy of the document with all sections, see nature.com/documents/nr-reporting-summary-flat.pdf

Life sciences study design

All studies must disclose on these points even when the disclosure is negative.

Sample size	For the experiments, we used a sample size of n >=3. This choice was based on similar experiments from previously reported studies (e.g., doi:10.1111/mmi.13379; DOI: 10.1098/rsob.180066). Due to the relatively low variability in the data, this sample size provided sufficient statistical power.
Data exclusions	No data were excluded from the analyses.
Replication	To ensure the reproducibility of our experimental findings, we implemented the following measures: (i) We took special care to preserve and maintain our samples. (ii) We meticulously applied and repeated protocols in a standardized manner. (iii) We incorporated suitable controls, including both positive and negative controls. (iv) We replicated each experiment at least three times. (v) We employed appropriate statistical analyses for accurate interpretation.<br><br>We were able to replicate the results for all different experiments included in the manuscript at least three times.
Randomization	Samples in the experiments were allocated randomly.
Blinding	Blinding was not relevant to our study. The outcomes of the experiments were objective, as they were derived from instruments, analyzed using scientific equipment and software, and were not influenced by researcher judgments.

Reporting for specific materials, systems and methods

We require information from authors about some types of materials, experimental systems and methods used in many studies. Here, indicate whether each material, system or method listed is relevant to your study. If you are not sure if a list item applies to your research, read the appropriate section before selecting a response.

Materials & experimental systems

n/a	Involved in the study
☐	☒ Antibodies
☒	☐ Eukaryotic cell lines
☒	☐ Palaeontology and archaeology
☒	☐ Animals and other organisms
☒	☐ Clinical data
☒	☐ Dual use research of concern
☒	☐ Plants

Methods

n/a	Involved in the study
☒	☐ ChIP-seq
☒	☐ Flow cytometry
☒	☐ MRI-based neuroimaging

Antibodies

Antibodies used

- A polyclonal rabbit serum against CsgA was used as the primary antibody. The polyclonal serum was kindly provided by Dr. Lynette Cegelski (Stanford University, US) and was manufactured by Proteintech Group Inc (US).

-A polyclonal rabbit serum against CsgD was used as the primary antibody. The polyclonal serum was kindly provided by Dr. Regine Hengge (Humboldt-Universität zu Berlin) and was produced by Pineda Antikörper-Service (Germany).

- A polyclonal goat antibody against Rabbit IgG conjugated with Alkaline Phosphatase was employed as the secondary antibody. This alkaline phosphatase-conjugated antibody is commercially available from Sigma (catalog number A3687; https://www.sigmaaldrich.com/AR/es/product/sigma/a3687?gclid=Cj0KCQjwvL-oBhCxARIsAHkOiu3M3YrL1csb6v0UUQaRXUf0KFxFH5OCEGoA97BRN4ziNCkH_BPy0XEaAskfEALw_wcB)

Validation

- The utilization of the polyclonal anti-CsgA serum was previously documented by Dr. Lynette Cegelski (doi.org/10.1371/journal.pone.0140388; doi.org/10.1038/nchembio.242).

- The application of the polyclonal anti-CsgD serum was previously described by Dr. Regine Hengge (doi.org/10.1111/mmi.13379; doi.org/10.1111/1462-2920.12991; doi.org/10.15252/emmm.201404309).

- The secondary antibody, goat anti-rabbit IgG alkaline phosphatase conjugate (Sigma A3687), is a well-established and extensively validated reagent in widespread use.
